# Nitric oxide activates intradomain disulfide bond formation in the kinase loop of Akt1/PKBα after burn injury

**DOI:** 10.3892/ijmm.2013.1241

**Published:** 2013-01-11

**Authors:** X.-M. LU, R.G. TOMPKINS, A.J. FISCHMAN

**Affiliations:** 1 Surgical Service, Massachusetts General Hospital, Boston, MA, USA;; 2 Harvard Medical School, Boston, MA, USA;; 3 Shriners Hospital for Children, Boston, MA, USA

**Keywords:** Akt1/protein kinase Bα, S-nitrosylation, disulfide bond, MS/MS

## Abstract

Severe burn injury is an acute inflammatory state with massive alterations in gene expression and levels of growth factors, cytokines and free radicals. During the catabolic processes, changes in insulin sensitivity and skeletal muscle wasting (unintended loss of 5–15% of lean body mass) are observed clinically. Here, we reveal a novel molecular mechanism of Akt1/protein kinase B*α* (Akt1/PKB*α*) regulated via cross-talking between dephosphorylation of Thr^308^ and S-nitrosylation of Cys^296^ post severe burn injury, which were characterized using nano-LC interfaced with tandem quadrupole time-of-fight mass spectrometry (Q-TOF)^micro^ tandem mass spectrometry in both *in vitro* and *in vivo* studies. For the *in vitro* studies, Akt1/PKB*α* was S-nitrosylated with S-nitrosoglutathione and derivatized by three methods. The derivatives were isolated by SDS-PAGE, trypsinized and analyzed by the tandem MS. For the *in vivo* studies, Akt1/PKB*α* in muscle lysates from burned rats was immuno-precipitated, derivatized with HPDP-Biotin and analyzed as above. The studies demonstrated that the NO free radical reacts with the free thiol of Cys^296^ to produce a Cys^296^-SNO intermediate which accelerates interaction with Cys^310^ to form Cys^296^-Cys^310^ in the kinase loop. MS/MS sequence analysis indicated that the dipeptide, linked via Cys^296^-Cys^310^, underwent dephosphorylation at Thr^308^. These effects were not observed in lysates from sham animals. As a result of this dual effect of burn injury, the loose conformation that is slightly stabilized by the Lys^297^-Thr^308^ salt bridge may be replaced by a more rigid structure which may block substrate access. Together with the findings of our previous report concerning mild IRS-1 integrity changes post burn, it is reasonable to conclude that the impaired Akt1/PKB*α* has a major impact on FOXO3 subcellular distribution and activities.

## Introduction

Metabolic alterations that are produced by critical illness such as burn trauma are associated with a hypermetabolic/inflammatory state, increased protein catabolism (with resulting muscle wasting) and insulin resistance. Muscle wasting can lead to muscle weakness that can result in hypoventilation, prolongation of dependence on mechanical ventilation, prolonged rehabilitation and even death (1–4). Insulin resistance is a well established state in critically ill patients and is considered to play a key role in the metabolic derangements and muscle wasting. Binding of insulin to its receptor (IR) activates IR tyrosine kinase, which then phosphorylates IR substrates (IRSs). Phosphorylation of IRS1 and IRS2 transduces the signal from IR to phosphatidylinositol-3-kinase (PI3-kinase) (4,5). Post-translational modifications (PTMs) of the insulin signaling system are considered to be major disease-dependent events that regulate glucose transport via GLUT-4 translocation and protein synthesis (6–12).

Akt1/PKB*α* is a critical downstream mediator of the IR/IRS/PI3-kinase pathway of the insulin signaling system (13–17). Akt1/PKB*α* consists of three structural features: the N-terminal pleckstrin homology (PH) domain, a large central kinase domain and a short C-terminal hydrophobic motif. High specific binding of the PH domain with membrane lipid products of PI3-kinase recruits Akt1/PKB*α* to the plasma membrane where phosphorylations of Thr^308^ (pThr^308^, kinase domain) and Ser^473^ (pSer^473^, hydrophobic motif) occur. Phosphorylation of Thr^308^ partially stimulates kinase activity; however, additional phosphorylation of Ser^473^ is required for full activity. Activation is associated with a disordered to ordered transition of a specific *α*C helix of Akt1/PKB*α* via an allosteric mechanism. A salt bridge between the side-chain of Lys^297^ and the phosphate group of pThr^308^ in this *α*C helix contributes to an ordered activation segment from ^292^DFG to APE^319^(18–21). Reversible dephosphorylations of Thr^308^ and Ser^473^ by protein phosphatase 2A (PP2A) and PH domain leucine-rich repeat protein phosphatase (PHLPP*α*) also occur in the Akt1/PKB*α* activation/deactivation cycle (22–25).

In addition to the role of reversible phosphorylation/dephosphorylation in the regulation of Akt1/PKB*α* activity, this kinase is also reversibly inactivated by S-nitrosylation under conditions that result in persistently increased production of nitric oxide; such as after burn injury (13,26–29). Thiol titration and NMR data indicate that a disulfide bond (Cys^60^-Cys^77^) exists in the kinase PH domain (30). A second disulfide bond in the critical kinase activation loop (Cys^297^-Cys^311^) has been reported to be associated with dephosphorylation under oxidative stress *in vitro*(31). In addition, it has been shown that when Cys^224^ of Akt1/PKB*α* is mutated to a Ser residue, the kinase becomes resistant to NO donor-induced S-nitrosylation and inactivation; suggesting that this residue is a major S-nitrosylation acceptor site (28). *In vivo* S-nitrosylations of the insulin receptor β and Akt1/PKB*α* result in reductions in their kinase activities (27). These data suggest that the redox status of Akt1/PKB*α*, regulated by NO, is a second factor in the PTM that modulates kinase activity (via dynamic conformational changes) and thus GLUT-4 trafficking and protein synthesis. Nevertheless, to date, published data on the reversible phosphorylation(s) and S-nitrosylation(s) relevant to Akt1/PKB*α* activation, conformation and regulation have not provided conclusive information concerning their interrelationships nor critical S-nitrosylation sites involved in the kinase activation/deactivation cycle.

Recent technical developments have made it feasible to study the molecular details of these important processes. These techniques include: i) sensitive and site-specific procedures for the detection of S-nitrosylation based upon nano-LC interfaced with tandem MS (32,33); ii) the Biotin-Switch method for qualitative discrimination of the thiol state between free, disulfide bonded and S-nitroylated cysteine residues under carefully defined conditions (34–39). Potential problems related to quantification with this technique have been discussed previously (33); and iii) highly specific anti-Akt1/ PKB*α* mAbs that can be used to immunoprecipitate quantities of protein that are sufficient to yield SDS-PAGE bands with Coomassie brilliant blue R-250 staining which are compatible with tandem MS analysis.

Burn injury-associated impairments in IRS1 signaling and attenuated IR-IRS-PI3K-Akt/PKB activation have been the major focuses of our research team (9,26,29,33). Significantly reduced phosphorylations of Ser^473^ and Thr^308^, as well as decreased Akt/PKB kinase activity were observed after burn injury [55% total body surface area (TBSA), day 3] and insulin stimulation (26). However, the interrelationship between impaired kinase activity and the loop disulfide bond (31) reported under oxidative stress remains unclear. In the present study we investigated the interaction between S-nitrosylation and phosphorylation at Cys^296^-Lys^297^ and Thr^308^-Phe^309^-Cys^310^ in the kinase loop at the proteomic level.

Specifically, the following issues need to be studied: i) the ability of Cys^296^ to chemically quench elevated levels of free radicals, mainly nitric oxide; ii) loop conformational changes associated with two types of PTMs; iii) quantitative proteomics of Akt1/PKBα by stable isotope labeling in mice. In this study, we obtained MS/MS sequence data to characterize the thiol states of Cys^296^ in the kinase activity loop of Akt1/PKB. These measurements were possible despite the extremely low level of nitrosylated protein (at the 10^−15^ pmol level, the chance of positive hits is ∼25% with lysates prepared from 25 mg of soleus muscle). The biochemical role of S-nitrosylation at Cys^296^ was characterized as an intermediate state which reduces the kinetic barrier to form the disulfide bond with Cys^310^ within the activity loop. This occurs simultaneously with dephosphorylation of pThr^308^ after burn injury. The facts that no other disulfide bonds associated with Cys^296^ were detected suggest that they may be thermodynamically forbidden; due to geometry and/or dihedral strain. The data obtained with soleus muscle from burned and sham-treated rats indicates that NO-mediated formation of the Cys^296^-Cys^310^ disulfide bond (which likely downregulates kinase activity) plays a reciprocal role with formation of a Lys^297^-pThr^308^ salt bridge (which upregulates kinase activity) during disease-associated reversible activation/deactivation processes.

## Materials and methods

### Chemicals

Acetonitrile (ACN, LC-MS Chromasolv), formic acid (FA), glacial acetic acid, LC-MS grade water, dithiothreitol (DTT), iodoacetic acid, iodoacetamide, [Glu^1^]-fibrinopeptide B, methyl methanethiolsulfonate (MMTS), S-nitrosoglutathione (GSNO), sodium L-ascorbate, neocuproine, N,N-dimethylformamide (DMF), dimethyl sulfoxide (DMSO) were obtained from Sigma Chemical Co. (St. Louis, MO). SDS-PAGE Ready gels (4-15% Tris-HCl, cat. no. 161-1122), Laemmli sample buffer (cat. no. 161-0737) and Coomassie brilliant blue R-250 (no. 161-0436) were obtained from Bio-Rad. Trypsin profile IGD kits (cat. no. PP0100) were obtained from Sigma. Anti-Akt1/PKB*α* monoclonal antibody (cat. no. 05-798; lot, 26860) and inactive Akt1/PKB*α* (cat. no. 14-279) were purchased from Upstate (Charlottesville, VA, USA). Streptavidin agarose CL-4B (cat. no. 85881) was a product of Fluka (Milwakee, WI, USA). HPDP-Biotin (cat. no. 21341) and Iodoacetyl-LC-Biotin (cat. no. 21333) were purchased from Pierce (Rockford, IL, USA).

### Mapping of cysteine residues in inactive Akt1/PKBα

Inactive Akt1/PKB*α* (10 μg, 0.18 nmol, in 10 μl stock solution) was transferred to a siliconized Eppendorf tube (0.6 ml) containing Laemmli sample buffer (2X, 10 μl, pH was adjusted to 8.0) and DDT (2 μl, 20 nmol, PBS, pH 8.0), and the solution was kept at 95°C for 5 min. Freshly prepared Iodoacetyl-LC-Biotin (15 μl, 55 nmol, in DMF) was added to the denatured protein solution followed by stirring for an additional 15 min at room temperature. The resulting biotinylated Akt1/PKB*α* was purified by SDS-PAGE and stained with Coomassie brilliant blue R-250. The protein bands were excised (∼1 mm size) and digested (Akt1/PKB*α*: trypsin 25, overnight at 37°C) with a Trypsin Profile IGD kit according to the manufacturer's instructions. The biotinylated peptide mixture was captured by gentle stirring with streptavidin agarose CL-4B (30 μl packed) at room temperature for 1 h (final vol, 100 μl). The streptavidin beads were washed with PBS (0.5 ml ×3), followed by water/ acetonitrile (ACN 10%, 0.5 ml ×3). Biotinylated peptides were released from the streptavidin beads with formic acid (70%, 100 μl) at room temperature for 15 min with brief vortexing. The supernatant containing biotinylated peptides was transferred to a new vial and the formic acid was evaporated with a SpeedVac. The biotinylated peptide mixture was resuspended in water/acetonitrile (ACN, 2%, with 0.1% FA, 70 μl), and the aliquots (10 μl) were injected into a Waters CapLC-tandem quadrupole time-of-fight mass spectrometry (Q-TOF) system.

### Identification of disulfide bonds in inactive Akt1/PKBα

Inactive Akt1/PKB*α* (10 μg, 0.18 nmol, in 10 μl stock solution) was transferred into a siliconized Eppendorf tube (0.6 ml) containing Laemmli sample buffer (2X, 10 μl, pH 8.0) and iodoacetamide (2 μl, 20 nmol, PBS, pH 8.0). The mixture was maintained at 95°C for 5 min and then stirred at room temperature for an additional 15 min. The Akt1/PKB*α* was purified by SDS-PAGE and stained with Coomassie brilliant blue R-250. The protein bands were processed as above.

### Identification of NO acceptor sites in inactive Akt1/PKBα

Three samples of inactive Akt1/PKB*α* (10 μg, 0.18 nmol, in 10 μl stock solution) were treated with GSNO (250 nmol, 50 μl PBS, pH 8.0, 200-fold excess/thiol group) for 1 h at room temperature in the dark in siliconized Eppendorf tubes (0.6 ml). Separation of Akt1/PKB*α* and GSNO was achieved by two successive acetone/water precipitations (0.3 ml, 70% ACN) at −40°C for 10 min. The supernatants (containing GSNO) were removed by centrifugation at 14,000 × g for 2 min. The kinase pellets were resuspended in blocking buffer (100 μl, 20 mM Tris-HCl, pH 7.7, 2.5% SDS, 20 mM MMTS, 1 mM EDTA, 0.1 mM neocuproine) at room temperature for 1 h with gentle stirring (1 mm ID ×5 mm bar). Excess MMTS was removed by acetone (100%, 0.3 ml) precipitation (as above), and the protein pellets were resuspended in PBS (50 μl, pH 8.0). Freshly prepared iodoacetic acid (5 μl, 2 mM in PBS, pH 8.0), HPDP-Biotin (5 μl, 2 mM in DMSO), Iodoacetyl-LC-Biotin (5 μl, 2 mM in DMF) and sodium ascorbate (20 μl, 5 mM, PBS) were added to the three vials containing nitrosylated Akt1/PKB*α*, respectively. The reaction mixtures were stirred at room temperature for 15 min (iodoacetic acid and Iodoacetyl-LC-Biotin) or 1 h for the thiol-disulfide exchange reaction. Aliquots of SDS sample buffer (2X, with 5% 2-mercaptoethanol, 50 μl) were added to the protein solutions, and the mixtures were incubated at 95°C for 5 min. The derivatized proteins were processed as above. Carboxymethyl cysteine (CMC)-containing peptides, were neutralized with FA (5 μl) and sequenced via parent ion discovery trigged by the CMC immonium ion (134.02±0.05 mDa) as reported previously (33). Biotinylated peptides were sequenced with data-dependent acquisition after capture with streptavidin agarose beads. Ten-microliter aliquots of each final solution were injected into the CapLC-Q-TOF system.

### Analysis of the Cys^296^-Cys^310^ disulfide bond formation in Akt1/PKBα after treatment with S-nitrosoglutathione

Inactive Akt1/PKB*α* (10 μg, 10 μl, 0.18 nmol) and freshly prepared GSNO (5 μl, 250 nmol, PBS, pH 8.0) were stirred in an Eppendorf tube (0.6 ml) in the dark at room temperature for 1 h. Separation of Akt1/PKB*α* and GSNO was performed with acetone/water (70%) as above. The kinase pellet was resuspended in PBS (10 μl), and SDS sample buffer (10 μl with iodoacetamide, 20 nmol) was added. The cysteine alkylation was performed at room temperature for 15 min. The protein samples were separated with SDS-PAGE Ready gels and digested as above. Aliquots of the final solution (10 μl) were injected into the CapLC-Q-TOF system.

### Measurement of the free and disulfide bonded Cys^296^ in Akt1/PKBα from soleus muscle of burned rats

Soleus muscle lysates from rats with third degree burn (40% TBSA) were prepared as previously described (29,33). The lysates (∼10 mg/ml total proteins) were diluted to ∼3-5 mg protein/ml protein with PBS, and filtered through 0.22-μm membranes. Immunoprecipitation was performed as follows. Anti-Akt1/PKB*α* mAb (clone AW24, 5 μg; Upstate) and prewashed protein G agarose beads (50 μl, packed) were kept at 4°C (100 μl of PBS) for 1 h under gentle stirring. Without washing the beads, the soleus lysates (5 ml) were added and stirring was continued for an additional 90 min. Non-specific proteins were removed by washing with PBS (3X), Laemmli sample buffer (50 μl, pH 8) containing HPDP-Biotin (400 μM) was added and the mixtures were maintained at 95°C for 5 min. The procedures for SDS-PAGE separation and in-gel trypsin digestion were the same as described above.

The burn injury protocol was approved by the Committee on Research Animal Care and Use of the Massachusetts General Hospital (MGH). The MGH animal care facility is accredited by the Association for Assessment and Accreditation of Laboratory Animal Care.

### LC-MS/MS analysis

All experiments were performed using a Waters CapLC-Q-TOF^micro^ system (Waters Corporation, Milford, MA, USA) as previously described (32,33). An analytical column (75 mm ID ×150 mm, C18 PepMap300, 5 mm, LC Packings) was used to connect the stream select module of the CapLC with the voltage supply adapter for ESI. Peptide mixtures were loading onto the precolumn (C18 resin) at a flow rate of 15 μl/min. Dead volume from the CapLC injector to the precolumn was measured to be ∼1.5 μl. After washing with mobile phase C (auxiliary pump, 0.1% formic acid in water/ACN, 2% ACN) for 2 min, the trapped peptides were back-washed from the precolumn onto the analytical column using the 10-position stream switching valve. Freshly prepared mobile phases A and C were sonicated under vacuum for ∼25 min, and mobile phase B was treated in this way for 5 min. The mobile phases were degassed every week, and the CapLC pumps were wet primed for 20 cycles. A linear gradient was used to elute the peptide mixture from mobile phase A (0.1% FA in water/ACN, 2% ACN) to mobile phase B (0.1% FA in ACN). The gradient was segmented as follow: isocratic elution with 2% B for 3 min, increasing B from 2 to 70% (3-40 min), isocratic elution with 70% B (40-45 min) and decreasing B from 70 to 2% (over 2 min). The injector syringe (25 μl) was washed with degassed mobile phase A, and the injection volume was set as full loop mode (10 μl). The gradient flow rate was set at 1.5 μl/min before the 16/1 Nanotee splitter and the pressure drop from the analytical column was ∼800 psi. The pressure drop (or the flow splitting ratio) was adjusted and maintained with 20 μm ID capillary tubing at the waste outlet position of the Nanotee splitter. The gradient flow rate was ∼95 nl/min. The electrospray voltage was set to ∼3,000 V to obtain an even ESI plume at the beginning of the gradient (high water content). As a routine sensitivity check, the PicoTip Emitter position and other parameters were adjusted to achieve ∼45 counts/sec for the capillary tubing background peak (m/z 429). Sample cone and extraction cone voltages were set at 45 and 3 V, respectively. The instrument was operated in positive ion mode with the electrospray source maintained at 90°C. The instrument was calibrated with synthetic human [Glu^1^]-fibrinopeptide B (100 fmol/μl in acetonitrile/water, 10:90, 0.1% formic acid, v/v) at an infusion rate of 1 μl/min in TOF MS/MS mode. The peptide was selected at m/z 785.8 and focused into the collision cell containing argon gas at ∼3×10^−5^ Torr; the collision energy was set at 35 V. Instrument resolution for the [Glu^1^]-fibrinopeptide B parent ion, m/z 785.84, was found to be 5,250 FWHM. All data were acquired and processed using MassLynx 4.1 software. For parent ion discovery triggered by the CMC immonium ion (134.02±0.03 Da), the survey low and high collision energies were set at 5 and 30 V, respectively. MS survey data were collected in continuum mode over the m/z 100–1,200 range. Data-dependent acquisition (DDA) was set from 450 to 1,500 m/z for the biotinylated peptides. Scan time was in the range of 1.9–3.8 sec (depending upon sample conditions), and the inter-scan delay was 0.1 sec. MS to MS/MS switch criteria were dependent upon the reporter ion intensity (5 counts/sec) and detection window (2.3 Da, charge status). The instrument was switched from MS/MS back to MS after 5 sec without intensity restriction.

### Evaluation of the S-nitrosylated cysteine site

Confirmations of the S-nitrosylation sites were performed by the following three step procedure. i) For parent ion discoveries by continuum MS survey, the peptide mass tolerance was 0.2 Da for the CMC immonium ion. Under these conditions, only a few false positive ions were observed and these were eliminated manually from the expected CMC parent ion list. ii) The positively discovered parent ions were analyzed with PepSeq of MassLynx V4.1 software; oxidation of methionine was searched as a variable modification. iii) For peptides, with MS/MS scores <35, manual interpretations of candidate parent ions were performed with the following procedure: continuum MS/MS spectra were smoothed, the upper 80% was centroided and cysteine residues were confirmed with three different thiol-specifically derivatized y ions. Cysteine residue monoisotopic mass C_3_H_5_NOS = 103.01 Da was replaced with CMC residue monoisotopic mass C_5_H_7_NO_3_S = 161.01 Da, HPDP-Biotin derivatized adduct residue monoisotopic mass C_22_H_37_N_5_O_4_S_3_ = 531.20 Da and Iodoacetyl-LC-Biotin derivatized adduct residue monoisotopic mass C_21_H_35_N_5_O_4S2_ = 485.21 Da, respectively.

## Results and Discussion

It has been reported that NO production is elevated by stressors such as burn injury and in patients with type 2 diabetes (29–41). It has also been shown that the Cys^297^-Cys^311^ disulfide bond in the critical kinase activation loop of Akt1/PKB*α* may be formed in association with dephosphorylation under oxidative stress *in vitro*(31). Thus, we hypothesized that reversible S-nitrosylation at either Cys^296^ or Cys^310^ in the kinase active loop may be a second PTM factor which complements reversible phosphorylation at Thr^308^ in the regulation of kinase activity and we sought to determine how S-nitrosylation interacts with phosphorylation during the Akt1/PKB*α* activation cycle (22). To address these issues, GSNO was used as the only NO donor in a model S-nitrosylation system to randomly target the seven cysteine residues of the kinase at pH 8. Vicinal Cys^296^ and Cys^310^ take advantage of the pKa for dissociation of the thiol to thiolate, and these electron-rich thiolate groups can lead to formation of an intradomain disulfide bond. Under these conditions, intracellular free cysteine residues, and cysteines at the kinase surface without interactions or located in hydrophobic environments (i.e. high pKa), are unlikely to be affected by GSNO. In contrast, Cys^296^ and Cys^310^, which may have low pKa values due to weak interactions with vicinal residues inside the loop, are potential S-nitrosylation sites as predicted from the 3D structure of the kinase (19). NO donors, such as thioredoxin and thiol/disulfide oxidoreductases were excluded from the system to prevent possible interferences (42,43); however, a small amount of 2-mercaptoethanol (∼0.05% v/v) was necessary to prevent oxygen effects.

The simple, but well-defined, S-nitrosylation reaction model was used to probe for particular NO acceptor sites in human Akt1/PKB*α* (inactive, 89% pure containing 2-mercaptoethanol and EGTA; Upstate) in three steps. i) Mapping of all cysteine residues with DTT reduction, Iodoacetyl-LC-Biotin alkylation and affinity capture provided relative MS ionization efficacies and charge states. ii) Detection of disulfide bonds with and without GSNO, provided an understanding of NO-mediated disulfide bond formation. The concentrations of the NO donor used here were similar to the levels used in reported studies (35–37). iii) MS/MS pinpointed the S-nitrosylated sites with three different thiol-specific derivatives. As indicated above, false-negatives may occur with the Biotin-Switch method (33), whereas false-positives are more common with the other methods; however, thiolether derivatives can be identified with MS/MS data. The findings of these studies were used to study the biological consequences of S-nitrosylation of Akt1/PKB*α* in soleus muscle from burned rats. This *in vivo* system was used because soleus muscle is an insulin-sensitive tissue with high levels of IRS-1.

**Figure 1.**
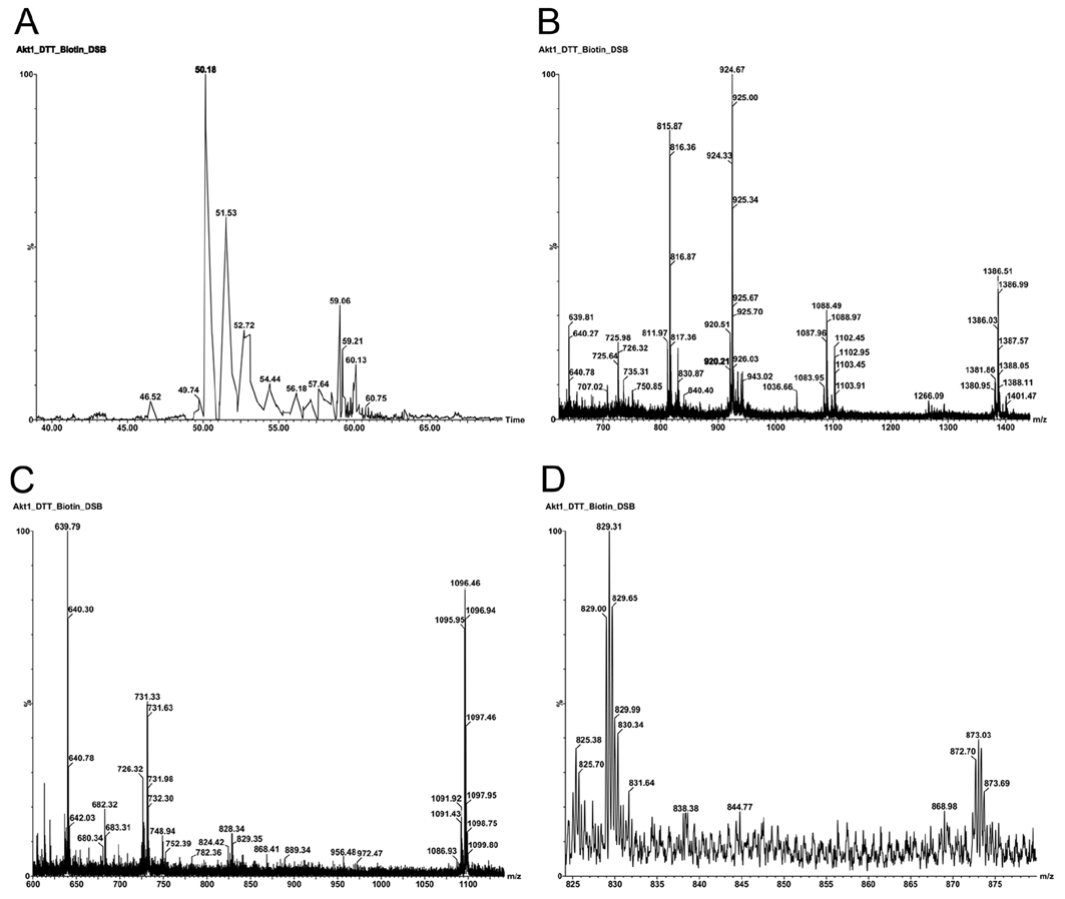
Mapping of cysteine residues in inactive Akt1/PKBα. (A) Base peak intensity (BPI) nano-LC chromatogram of affinity capture of all seven cysteine residues that were biotinylated with Iodoacetyl-LC-Biotin. Sample preparation: see materials and methods section for details. Column conditions: 75 mm ID ×150 mm, C18 PepMap300, 5 mm, under linear gradient conditions at a flow rate 95 nl/min. (B) TOF MS analysis of parent ions co-eluted at retention time of ∼53 min. Parent ions m/z 924.67 and 1386.51 are triply and doubly charged ions from the same tryptic peptide ^308^TFCGTPEYLAPEVLEDNDYGR^328^ which contains Cys^310^. Parent ion m/z 1266.09 is a triply charged ion from the tryptic peptide, ^437^YFDEEFTAQMTITPPDQDDSMECVDSER^465^, which contains Cys^460^. Parent ion m/z 815.87 is a doubly charged ion derived from the tryptic peptide, ^77^CLQWTTVIER^86^, which contains Cys^77^. The parent ion at m/z 1088.49 results from CH_4_ neutral loss from m/z 1096.46 as shown in C. (C) TOF MS analysis of parent ions co-eluting at retention time of ∼50.8 min. Parent ions m/z 731.33 and 1096.46 are triply and doubly charged ions from the same tryptic peptide, ^49^ESPLNNFSVAQCQLMK^64^, which contains Cys^60^. Parent ion m/z 639.79 is doubly charged and is derived from tryptic peptide, ^290^ITDFGLCK^297^, which contains Cys^296^. (D) TOF MS analysis of parent ions co-eluting at retention time of ∼53.5 min. Parent ion m/z 829.00 is triply charged and derived from tryptic peptide, ^329^AVDWWGLGVVMYEMMCGR^346^, which contains Cys^344^. Parent ion m/z 872.70 is triply charged and derived from tryptic peptide, ^223^LCFVMEYANGGELFFHLSR^241^, which contains Cys^224^.

A base peak intensity (BPI) nano-LC chromatogram of all seven affinity captured cysteine residues that were biotinylated with Iodoacetyl-LC-Biotin is shown (Fig. 1A). Cysteine residue monoisotopic mass of C_3_H_5_NOS = 103.01 Da was replaced with derivatized Cys residue monoisotopic mass of C_21_H_35_N_5_O_4_S_2_ = 485.21 Da. The relative simplicity of the nano-LC chromatogram indicates the high purification efficacy for removing non-biotinylated tryptic peptides from streptavidin agarose beads. Three predominate TOF MS tryptic parent ions were identified; m/z 639.79 (T41, M+2H^+^ = 639.83) eluting at 50.5 min, m/z 1088.49 (T9, M-CH_4_+2H^+^ = 1088.03) eluting at 51.5 min and m/z 924.67 (T44, M+3H^+^ = 924.43) eluting at 53 min are doubly and triply charged tryptic peptides containing Cys^296^, Cys^310^ and Cys^60^, respectively. Fig. 1B shows the parent ions co-eluting at ∼53 min as well as the charge state assignments. Parent ions m/z 924.67 (T44, M+3H^+^ = 924.43) and m/z 1386.51 (T44, M+2H^+^ = 1386.14) are triply and doubly charged ions from the same tryptic peptide, ^308^TFCGTPEYLAPEVLEDNDYGR^328^, which contains Cys^310^. Parent ion m/z 1266.09 (T58, M+3H^+^ = 1266.41) is triply charged and derived from the peptide, ^437^YFDEEFTAQMTITPPDQDDSMECVDSER^465^, which contains Cys^460^. Parent ion m/z 815.87 (T11, M+2H^+^ = 815.93) is doubly charged from the peptide, ^77^CLQWTTVIER^86^, which contains Cys^77^. Parent ion m/z 1088.49 resulted from CH_4_ neutral loss from m/z 1096.48. Fig. 1C shows TOF MS parent ions that co-eluted at ∼50.8 min; chromatographic peak tailing the most intense peak at 50.5 min. Parent ions m/z 731.33 (T9, M+3H^+^ = 731.03) and m/z 1096.46 (T9, M+2H^+^ = 1096.04) are triply and doubly charged ions from the same tryptic peptide, ^49^ESPLNNFSVAQCQLMK^64^, which contains Cys^60^. Parent ion m/z 639.79 (T41, M+2H^+^ = 639.83) is a doubly charged ion from the tryptic peptide, ^290^ITDFGLCK^297^, which contains Cys^296^. Fig. 1D shows the TOF MS parent ions that co-eluted at ∼53.5 min. Parent ion m/z 829.00 (T45, M+3H^+^ = 829.05) is triply charged and derived from the tryptic peptide, ^329^AVDWWGLGVVMYEMMCGR^346^, which contains Cys^344^. Parent ion m/z 872.70 (T32, M+3H^+^ = 872.43) is triply charged and derived from the tryptic peptide, ^223^LCFVMEYANGGELFFHLSR^241^, which contains Cys^224^. No doubly charged T58, T45 or T32 ions were observed. It is clear that the ionization efficacies for the peptides containing Cys^296^ (M+2H^+^), Cys^310^ (M+2H^+^ and M+3H*+*), Cys^60^ (M+2H^+^ and M+3H^+^) and Cys^77^ (M+2H^+^) are much higher than for the triply charged peptides containing Cys^460^ (M+3H^+^), Cys334 (M+3H^+^) and Cys^224^ (M+3H^+^) under the same conditions.

**Figure 2.**
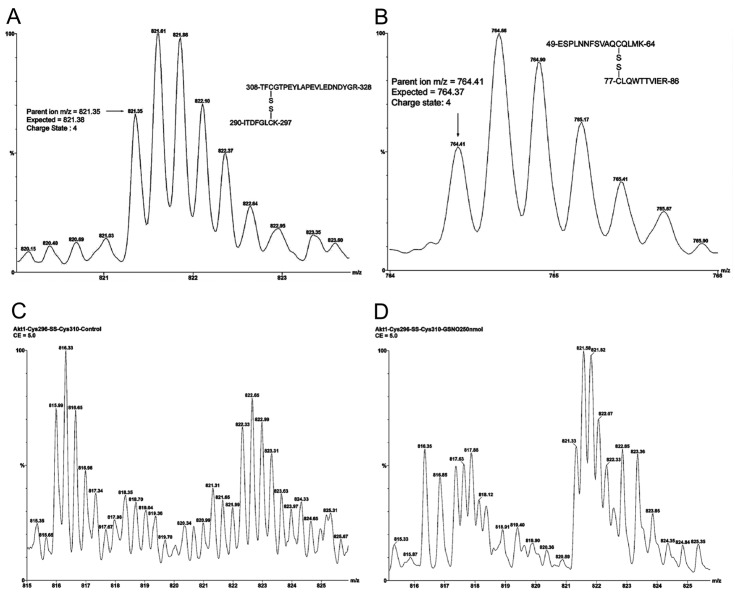
Detections of two intradomain disulfide bonds in Akt1/PKBα. (A) Detection of intradomain Cys^296^-Cys^310^ disulfide bond in the kinase loop. Inactive Akt1/PKBα (10 µg) was treated with GSNO and iodoacetamide (50 µM) in Laemmli sample buffer as in D. In-gel trypsin digestion was performed after SDS-PAGE separation (4–15% Tris-HCl). Monoisotopic parent ion at m/z 821.35, charge state 4. Expected quadruply charged disulfide linked Cys^296^ and Cys^310^ containing the peptide at m/z 821.38. (B) Detection of the intradomain Cys^60^-Cys^77^ disulfide bond in the PH domain. Monoisotopic parent ion at m/z 764.41, charge state 4. Expected quadruply charged disulfide bond linked Cys^60^ and Cys^77^-containing peptide at m/z 764.37. (C) Free thiol state of Cys^310^ in the kinase loop without NO donor. The triply charged parent ion m/z 815.99: ^308^TFCGTPEYLAPEVLEDNDYGR^328^ (expected: m/z 816.03, CAM derivative) represents the completely free thiol state of Cys^310^, while the triply charged m/z 821.31 is not from disulfide linked Cys^296^-Cys^310^ dipeptides (expected charge state 4). The Cys^296^-Cys^310^ disulfide bond was not detected in the absence of the NO donor. (D) Nitric oxide promotes the formation of the Cys^296^-Cys^310^ disulfide bond in the kinase loop. Inactive Akt1/PKBα (10 µg) was treated with GSNO (250 nmol, 50 μl PBS, pH 8.0, 1 h at room temperature in dark) prior to alkylation with iodoacetamide and SDS-PAGE. The doubly charged m/z 816.35 ion is not from a Cys^310^-containing tryptic peptide (expected charge state 3), and quadruply charged m/z 821.33 occurs at the expense of diminished triply charged Cys^310^ peptide. The free thiol of Cys^310^ is completely converted into the disulfide bond with Cys^296^.

When Akt1/PKB*α* was treated with GSNO without cleavage of disulfide bonds and the free cysteine residues were alkylated with iodoacetamide, two intradomain disulfide bonds were identified: Cys^60^-Cys^77^ in the PH domain and Cys^296^-Cys^310^ in the kinase active loop. The monoisotopic parent ion with m/z 821.35, shown in Fig. 2A, represents two tryptic peptides containing the Cys^296^-Cys^310^ disulfide bond in the kinase loop. The isotopic peaks at m/z 821.61 and m/z 821.35 are attributed to the M+1 and M+0 ions. A mass difference of 0.26 Da (expected 0.25 Da) indicated four positive charges: two at N-terminals and two at side chains of the C-terminals of the dipeptides. The expected quadruply charged disulfide bond linked Cys^296^ and Cys^310^-containing peptides (T41-SS-T44, M+4H^+^) were calculated to be m/z 821.38 [(894.45 + 2387.06 + 4)/4]. The monoisotopic parent ion with m/z 764.41, shown in Fig. 2B, represents the two tryptic peptides containing the Cys^60^-Cys^77^ disulfide bond in the PH domain. The quadruply charged state is calculated as m/z 764.66 (M+1) - 764.41 (M+0) = 0.25 which indicates four positive proton charges. The quadruply charged disulfide bond linked Cys^60^ and Cys*77* containing peptides (T9-SS-T11, M+4H^+^) are calculated as m/z 764.37 [(1806.86 + 1246.63 + 4)/4]. Without GSNO treatment, only the Cys^60^-Cys^77^ disulfide bond was detected. The mass accuracies for the two measurements were found to be 36 ppm (Cys^296^-Cys^310^ disulfide bond linked dipeptides) and 78 ppm (Cys^60^-Cys^77^ disulfide bond linked dipeptides). The impact of GSNO on Cys^296^-Cys^310^ disulfide bond formation is demonstrated in Fig. 2C and D. The S-nitrosylation reaction without GSNO (Fig. 2C) shows the triply charged tryptic peptide, ^308^TFCGTPEYLAPEVLEDNDYGR^328^, [carboxyamidomethyl cysteine (CAM) derivative] containing Cys^310^ at m/z 815.99 (expected monoisotopic parent ion, 816.03). The observed M+1 isotopic peak was at m/z 816.33. The difference between the isotopic M+1 and M+0 peak of 0.34 Da indicates three proton charges. In contrast, the triply charged ions at m/z 821.31 and 821.65 (difference = 0.31 Da) do not represent the quadruply charged Cys^296^-Cys^310^ dipeptides in Fig. 2C. The triply charged Cys^310^-containing peptide was found to be totally absent with GSNO treatment as shown in Fig. 2D. The doubly charged ions at m/z 816.35 and 816.85 (difference = 0.50 Da) are not related to the triply charged tryptic peptide ^308^TFCGTPEYLAPEVLEDNDYGR^328^ (CAM derivative) containing Cys^310^ at m/z 815.99 as shown in Fig. 2C. In contrast, the ions at m/z 821.33 and 821.58 (difference = 0.25 Da) are indeed from quadruply charged Cys^296^-Cys^310^-linked dipeptides. Since quadruply charged Cys^296^-Cys^310^-linked dipeptides are formed at the expense of triply charged Cys^310^-containing peptide after GSNO treatment, it is obvious that S-nitrosylation and disulfide bond formation occur simultaneously in the kinase loop.

We next sought to determine which cysteine residue is the NO acceptor that initializes Cys^296^-Cys^310^ disulfide bond formation. There are three possibilities for the two cysteine residue thiol states: single S-nitrosothiol, double S-nitrosothiols and nitroxyl disulfide. The last case (nitroxyl disulfide) can be ruled out from the list, since the expected net mass increases of 28 Da (NO - 2H = 30 - 2 Da) were not observed for the corresponding dipeptides. The second case, double S-nitrosothiols of Cys^296^ and Cys^310^, may occur if both pKa values are acidic inside the kinase loop. The Biotin-Switch method was used to identify the S-nitrosothiol within the loop under gentle reaction conditions (GSNO 250 nmol, 1 h). In addition, two other thiol-specific reagents, iodoacetic acid and Iodoacetyl-LC-Biotin (leaving molecule: HI, fast and quantitative), were evaluated.

Table I shows the expected results of Cys^296^ S-nitrosylation in the kinase loop with the three different chemical modifications. The resulting S-nitrosylated Cys was reduced with ascorbate and then derivatized with iodoacetic acid to afford the CMC derivative (the Cys residue with a monoisotopic mass C_3_H_5_NOS = 103.01 Da was replaced by the CMC residue with a monoisotopic mass C_5_H_7_NO_3_S = 161.01 Da) for sequence analysis. The CMC derivative of the y2 ion of the doubly charged tryptic peptide, ^290^ITDFGLCK^297^, was confirmed at m/z 308.17 (expected 308.13 = 161.01 + 145.10+ 2.02). The Cys HPDP-Biotin adduct (Cys residue monoisotopic mass C_3_H_5_NOS = 103.01 Da was replaced with the adduct residue monoisotopic mass C_22_H_37_N_5_O_4_S_3_ = 531.20 Da) was used for sequence analysis. The corresponding y2 ion of the Biotin-HPDP derivatized, ^290^ITDFGLCK^297^, was confirmed at m/z 678.29 (expected 678.32 = 531.20 + 145.10 + 2.02). The Cys Iodoacetyl-LC-Biotin adduct (Cys residue monoisotopic mass C_3_H_5_NOS = 103.01 Da was replaced with adduct residue monoisotopic mass C_21_H_35_N_5_O_4_S_2_ = 485.21 Da) was used for peptide sequence analysis. The corresponding y2 ion of Iodoacetyl-LC-Biotin derivatized, ^290^ITDFGLCK^297^ was confirmed at m/z 632.38 (expected 632.33 = 485.21 + 145.10 + 2.02). Since the y2 ions of ^296^Cys-Lys^297^ produced with the three different derivatization procedures were unambiguously observed it is likely that Cys^296^ is a favorable S-nitrosylation site under the conditions used. Although studies with mutated Akt1/PKB*α* (Cys^224^) indicated that Cys^224^ is a major S-nitrosylation acceptor site *in vitro*(28), the biological role of S-nitrosylated Cys^224^ in kinase regulation needs to be further explored. In the current study it was determined that significant S-nitrosylation of Cys^224^ is improbable, since using the three alkylation approaches and trypsin digestion, the levels of positive ionization of Cys^224^-containing peptides were below the level of detection. This failure in detection of S-nitrosylated Cys^224^ may be a false-negative under our experimental conditions and clearly warrants further investigation. Nevertheless, our findings clearly demonstrate that S-nitrosylated Cys^296^ is directly relevant to the kinase activation regulation cycle.

**Figure 3.**
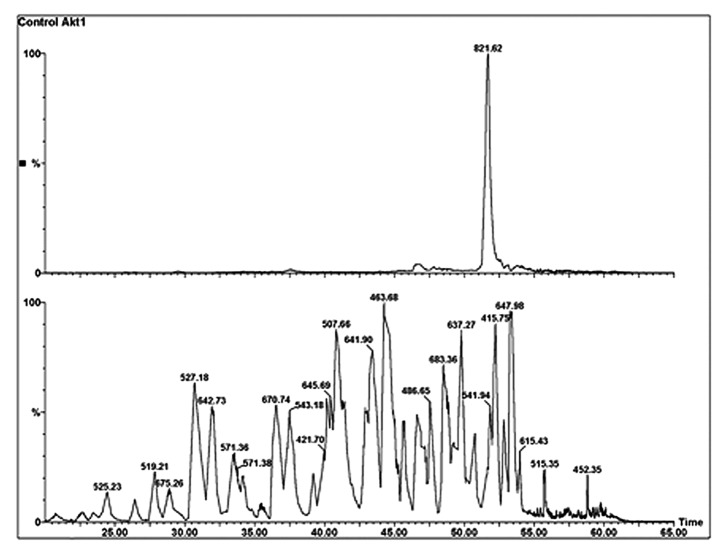
Nano-LC chromatogram of tryptic peptides of Akt1/PKBα and MS ion 821.62 chromatogram of soleus muscle. Top panel: mass ion chromatogram of the dipeptides m/z 821.62: M+1 isotopic peak of the quadruply charged dipeptides (intensity of M+0 monoisotopic peak is lower than M+1). Bottom panel: BPI chromatogram of the Akt1/PKBα tryptic peptides after immunoprecipitations and in-gel digestion from nano-LC interfaced with Q-TOF tandem mass spectrometry.

One possible explanation for the kinetics of Cys^296^-Cys^310^ disulfide bond formation in the kinase loop may be that there is a high kinetic barrier without GSNO. Due to its highly labile nature (44), S-nitrosylated Cys^296^, which forms rapidly in the presence of GSNO, may function as an intermediate state. Since this intermediate is likely to have a lower kinetic barrier for Cys^296^-Cys^310^ disulfide bond formation, the overall speed of the reaction should increase greatly. It has been reported that *trans*-nitrosylation reactions between vicinal thiols can occur and accelerate disulfide bond formation (45). The well characterized Cys^296^-Cys^310^ disulfide bond can be used as a signature peptide for detection of S-nitrosylation of Cys^296^ after immunoprecipitation. The separation of tryptic peptide mixtures with our nano-LC interfaced Q-TOF^micro^ is demonstrated in Fig. 3 (bottom panel). The extracted mass ion peak m/z 821.62, as shown in Fig. 3 (top panel), is the M+1 isotopic peak of the quadruply charged dipeptides (the most intense isotopic peak due to a high number of carbon atoms).

**Figure 4.**
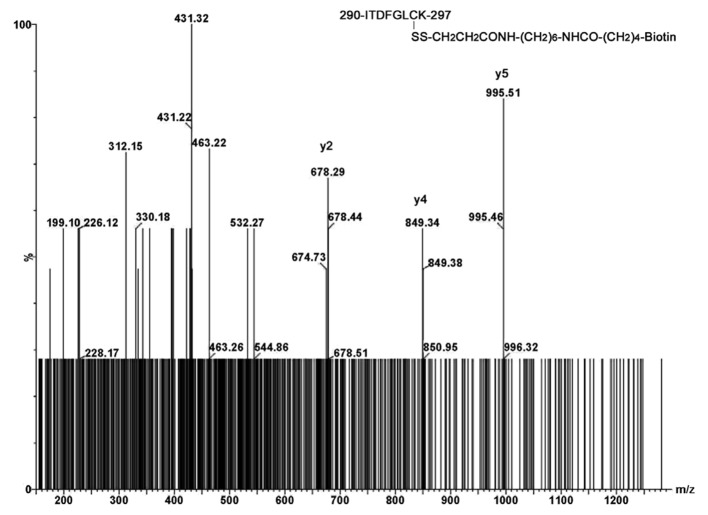
MS/MS sequence analysis of biotinylted free Cys^296^ peptide of Akt1/PKBα after burn injury. Rat soleus muscle lysates (30 mg total protein) were treated with anti-Akt1/PKBα mAb and in-gel biotination was performed with HPDP-Biotin. Parent ion m/z 662.84 (M+2H^+^, expected 662.82) was sequenced. Cys residue monoisotopic mass C3H_5_NOS = 103.01 Da is replaced with the adduct residue monoisotopic mass C22H_37_N_5_O_4_S_3_ = 531.20 Da. A low sequence score 18 was obtained from the parent ion with S/N = 3; however the critical diagnostic y2, y4 and y5 ions at m/z 678.29, 849.34 and 995.51 confirmed that trace amounts of free Cys^296^ are present after burn injury.

**Figure 5.**
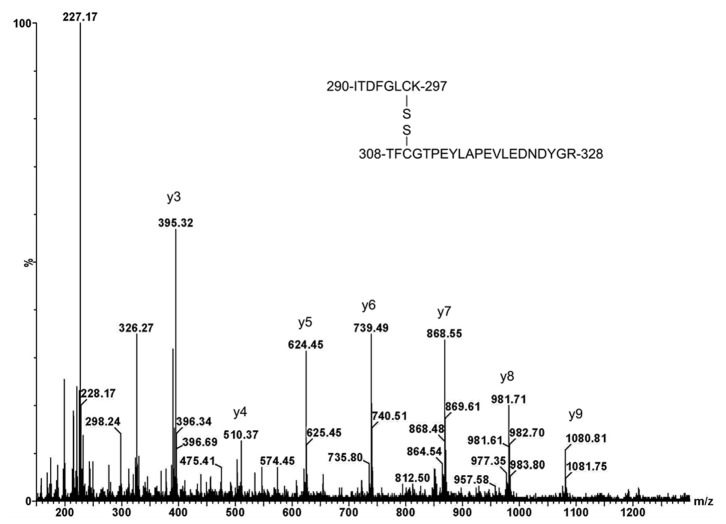
MS/MS sequence analysis of the Cys^296^-Cys^310^ disulfide-linked peptide with dephosphoryated Thr^308^ in soleus muscle from burned rats. Partially sequenced Cys^296^-Cys^310^ disulfide-linked dipeptides: C-terminal y ion series (y3 to y9) of Cys^310^-containing peptide, ^308^TFCGTPEYLAPEVLEDNDYGR^328^, were observed from the quadruply charged parent ion (T41-SS-T44, M+4H^+^).

**Figure 6.**
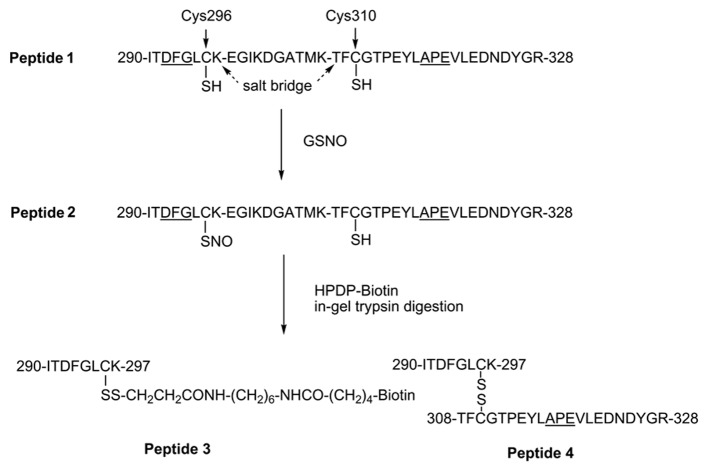
Proposed mechanism for Akt1/PKBα kinase regulation by phosphorylation and S-nitrosylation in the muscle of burned rats. Phosphorylation of Thr^308^ stabilizes the disordered loop structure between 292DFG and APE^319^ via a salt bridge with Lys^297^ as illustrated in the loop Peptide 1, which upregulates Akt1/PKBα kinase activity. NO free radical production is increased after burn injury. A large portion of Cys^296^ undergoes S-nitrosylation at Cys^296^ (Peptide 2); however, some free Cys^296^ remains (Peptide 3). S-nitrosylation activates Cys^296^-Cys^310^ intradomain disulfide bond formation (Peptide 4). S-nitrosylation at Cys^296^ is associated with dephosphorylation of Thr^308^ and inaccessibility to the kinase site; which downregulates kinase activity.

The *in vitro* system allowed us to determine conditions that are favorable for evaluation of S-nitrosylation of Cys^296^ by MS/MS and was useful for studying the mechanism of intradomain disulfide bond formation. The reason for using inactive Akt1/PKB*α* (unphosphorylated) in these studies was to find possible S-nitrosylation sites in relationship with the following published data: i) Akt1/PKB*α* undergoes transient phosphorylation/dephosphorylation which regulates the kinase activity conformation cycle (22); ii) kinase disulfide bond formation, Cys^297^-Cys^311^, and dephosphorylation at pThr^308^ are induced simultaneously by H*2*O_2_ oxidative stress *in vitro*(31); iii) high levels of nitric oxide production occur both after burn injury (29,42) and in diabetic patients (43). Previous results from our laboratory have indicated that there is S-nitrosylation at Cys^296^ in rat soleus muscle (33). A parent ion at m/z 690.83 containing Cys^296^ (T41-T42: ^290^ITCFGLCKEGIK^301^) was observed with CAM immonium trigged parent ion discovery; however, MS/MS sequencing data were not obtained. As a continuation of these studies to explore S-nitrosylation in the kinase active loop, large amounts of rat soleus muscle lysate (∼3-5 mg/ml total proteins, 3 ml for each experiment, day 4 after 40% TBSA, 3rd degree burn) were used. In the present study, detailed MS/MS analyses of HPDP-biotinylated free Cys^296^ peptide and Cys^296^-Cys^310^ disulfide bound dipeptides of Akt1/PKB*α* were performed with lysates of rat soleus muscle after burn injury. The tryptic parent ion derivatized from free Cys^296^ after burn injury was observed at m/z 662.84 (M+2H^+^, expected 662.82) and the MS/ MS sequence data are shown in Fig. 4. A low sequence score of 18 was obtained from the parent ion with S/N = 3. However, the critical diagnostic y2, y4 and y5 ions at m/z 678.29, 849.34 and 995.51 confirmed that trace amounts of free Cys^296^ are indeed present after intradomain disulfide bond formation induced by burn injury. In addition, partial sequencing data for Cys^296^-Cys^310^ disulfide-linked dipeptides are shown in Fig. 5. The C-terminal y ion series of Cys^310^-containing peptide, ^308^TFCGTPEYLAPEVLEDNDYGR^328^, was observed for the quadruply charged parent ion (T41-SS-T44, M+4H^+^). Cys^296^-Cys^310^ disulfide-linked dipeptides were not observed in muscle lysates from sham-treated animals (negative controls). The chance of obtaining the MS/MS sequence using our *in vivo* experimental conditions is only ∼20–25%. This indicates that one interpretable MS/MS outcome (score >25) is expected in four or five independent experiments in which three successive injections are performed. Nevertheless, these MS/MS data for peptides containing free Cys^296^ and Cys^296^-Cys^310^-linked dipeptides are sufficient to verify our hypothesis that S-nitrosylation promotes intradomain disulfide bond formation and dephosphorylation at pThr^308^ after burn injury as illustrated in Fig. 6. Due to its high lability of Cys^296^-SNO, direct identification of this species *in vivo* was not possible.

S-nitrosylation of Akt1/PKB*α* is a key factor for understanding the regulation of glucose transport and downstream protein synthesis. A recent study demonstrated that blockade of iNOS prevents the S-nitrosylations of Akt and IRS-1 and results in insulin resistance *in vivo*(46). Although it is clear that two PTMs of Akt1/PKB*α*, phosphorylation at Thr^308^ and S-nitrosylation at Cys^296^, are critical for the regulation of Akt1/PKB*α* activity under stress conditions, there are still many unanswered questions concerning how reversible phosphorylation/dephosphorylation and S-nitrosylation/denitrosylation modulate Akt1/PKB*α* activity. For example, it has been reported that the Cys^296^-Cys^310^ disulfide bond is present only when there is binding of substrate to the active kinase loop and phosphorylation at Thr^308^(25); indicating that both disulfide bond formation as well as phosphorylation of Thr^308^ are important for kinase activity. In contrast, this disulfide bond was not observed under similar conditions in two studies of the ternary structure of the kinase (19,21); even though, oxidative stress was shown to induce dephosphorylation of pThr^308^ and disulfide bond formation in the kinase loop in an *in vitro* study (31).

In summary, our data establish that Cys^296^ is an important S-nitrosylation site in the kinase loop of Akt1/PKB*α* under gentle reaction conditions: i) iodoacetic acid as previously described; ii) the HPDP-Biotin switch method; and iii) the Iodoacetyl-LC-Biotin method to ensure indirect capture of Cys^296^-SNO which may be undetectable with HPDP-Biotin. The corresponding derivatized y2 ions (^296^Cys-Lys^297^) in the tryptic peptide (Ile-Thr-Asp-Phe-Gly-Leu-Cys-Lys) were obtained with mass sequences to eliminate false-positive discovery. Although no other S-nitrosylated cysteine residues were detected, it is possible that S-nitrosylations at Cys^224^, Cys^344^ and Cys^460^ were missed due to very low ionizations (i.e., false-negative discoveries). As a consequence of S-nitrosylation at Cys^296^, there is rapid disulfide bond formation with vicinal Cys^310^ in the kinase loop, which alters kinase substrate recognition (47) as well as Akt-FOXO switch (48). This affords a stable disulfide bond linked quadruply charged parent ion at m/z 821.35 (M+4H^+^). Partial sequencing data for Cys^296^-Cys^310^ linked dipeptides from soleus muscle lysates indicated that burn injury is associated with both dephosphorylation of pThr^308^ and disulfide bond formation. These two types of PTMs may provide insights for understanding negative cooperative effects on reduced Akt/PKB kinase activity after burn injury as previously reported by our laboratory (26). Although our results have provided important mechanistic information, quantitative measurements of Thr^308^/pThr^308^ and free Cys^296^/ SNO-Cys^296^/bound Cys^296^ in patients with burn injury and type 2 diabetes remain very challenging.

## Figures and Tables

**Table I.** Characterization of the thiol-specifically modified Akt1/PKBα peptide ^290^ITDFGLCK^297^.

Chemical derivatives	Parent calc.	Parent found	y2 ion calc.	y2 ion found
CMC	953.45	953.42	308.13	308.17
HPDP-Biotin	1323.64	1323.68	678.32	678.29
Acetyl-LC-Biotin	1277.65	1277.58	632.33	632.38

## Abbreviations:

Akt1/PKBα: Akt1/protein kinase Bα;
CAM: carboxyamidomethyl cysteine;
CMC: carboxymethyl cysteine;
GSNO: S-nitrosoglutathione;
PH: pleckstrin homology;
PTM: post-translational modification;
Q-TOF: tandem quadrupole time-of-fight mass spectrometry;
TBSA: total body surface area
